# Menthol in the Treatment of Urticaria and Pruritus

**Published:** 1886-03

**Authors:** 


### Menthol in the Treatment of Urticaria and Pruritus.

Among the myriad of remedies for these troublesome affections we have no other which affords such complete and instantaneous relief as a solution of menthol. We have used this remedy for urticaria in three cases. Not only is the itching relieved for the time, but a cure seems to be affected. In pruritus ani, and in eczema, moistening the parts with menthol solution causes an immediate cessation of the pain. The solution should contain from two to ten grains of menthol to the ounce of water.

---

We are glad to welcome home again our friends, Dr. Frank H. Potter and Dr. DeLancy Rochester, from a six months' sojourn in Europe. They have spent the time pleasantly, and no doubt most profitably, in Berlin. Dr. Potter has devoted his time to the study of the practical workings of the microscope, and especially to clinical research in diseases of the lungs and internal organs. Dr. Rochester has continued his study in diseases of the throat and nose. We are sure they have added much to their already liberal education, and wish them the success which they richly deserve.

---

An interesting account of the Proceedings of the Medical Society of the State of New York has been crowded out of this number.

